# Improvement in the Quality of Early Postoperative Course After Endoscopic Transsphenoidal Pituitary Surgery: Description of Surgical Technique and Outcome

**DOI:** 10.3389/fneur.2020.527323

**Published:** 2020-10-20

**Authors:** Xinfa Pan, Yuehui Ma, Minwei Fang, Jiajing Jiang, Jie Shen, Renya Zhan

**Affiliations:** Department of Neurosurgery, The First Affiliated Hospital, School of Medicine, Zhejiang University, Hangzhou, China

**Keywords:** pituitary adenoma, endoscopic transsphenoidal surgery, enhanced recovery after surgery (ERAS), surgical technique, day surgery, nasal ventilation

## Abstract

**Objective:** The endoscopic transsphenoidal pituitary surgery has gained popularity and has shown excellent results with a more comfortable postoperative course. However, the quality of the early postoperative course is not well-established in endoscopic transsphenoidal pituitary surgery. We hypothesized that the quality of the early postoperative course would be improved when an enhanced recovery after surgery (ERAS) protocol and minimally invasive endoscopic transsphenoidal pituitary surgery is implemented.

**Methods:** We implemented a perioperative management ERAS protocol for endoscopic transsphenoidal pituitary surgery by an experienced surgeon (Yuehui Ma) in our department from January 2018. From then the endoscopic transsphenoidal pituitary surgery was implemented with a minimally invasive technique, such as bony sella reconstruction and partial nasal packing. We compared the results of 78 endoscopic transsphenoidal pituitary surgery cases during the initiation of the ERAS protocol and minimally invasive technique implementation: 37 cases in the control group and 41 cases in the ERAS group. Outcomes assessed included the effectiveness and security of surgery, postoperative hospital length of stay (LOS), and postoperative status on postoperative day 1 (POD1).

**Results:** Postoperative status on POD1, such as nasal ventilation, out of bed, headache score, and liquid supplement, had significant improvement (*P* < 0.05). The median postoperative LOS decreased from 8 days in the control group to 3 days in the ERAS group (*P* < 0.05). The ERAS group had better economic benefit with fewer hospital charges (*P* < 0.05). There was no difference in the early postoperative diabetes insipidus and 30-day readmission for epistaxis, hyponatremia, or other complications between the two groups.

**Conclusion:** The quality of the early postoperative course was improved when a neurosurgical ERAS protocol and minimally invasive endoscopic transsphenoidal pituitary surgery with partially nasal packing were implemented. Endoscopic transsphenoidal pituitary day surgery could be recommended in some classes of patients though further evaluation in large case studies is warranted.

## Introduction

Pituitary adenomas (PAs) are the most common benign neoplasms in the sellar region, and surgery by the transsphenoidal approach remains the best choice for a majority of PAs (1, 2). With the progress of the endoscopic concept in nasal sinus surgery and new improvements in endoscope equipment, the endoscopic transsphenoidal approach has gradually become the mainstream surgical method worldwide (3–5). The preservation of sinonasal function, less bleeding, and a comfortable postoperative course have all been considered as advantages of the endoscopic transsphenoidal surgery. Additionally, multiple studies have shown shorter postoperative hospital length of stay (LOS) for patients undergoing endoscopic transsphenoidal pituitary surgery (6). However, these studies focused on the results of surgical procedures, and only a few clinical researches report the quality of the early postoperative course after endoscopic transsphenoidal pituitary surgery (7, 8).

LOS is only an indirect indicator of postoperative comfort. The quality of the early postoperative course is the key to determining LOS, early surgical results, and even long-term prognosis (9). Patient condition on postoperative day 1 (POD1), including nasal ventilation, headache score, out of bed, and liquid supplement, can represent the quality of the early postoperative course after endoscopic transsphenoidal pituitary surgery. Minimally invasive endoscopic pituitary surgery and a perioperative management protocol for the care of patients are the two important points to facilitate a short LOS, safe early discharge, and a more comfortable postoperative course (10).

Here, we describe our experience with the implementation of a perioperative management enhanced recovery after surgery (ERAS) protocol for minimally invasive endoscopic transsphenoidal pituitary surgery at a regional center hospital in China. The purpose of our study is to assess the quality of the early postoperative course and to describe the surgical technique during minimally invasive endoscopic transsphenoidal pituitary surgery.

## Materials and Methods

### Perioperative Management ERAS Protocol and Conventional Care

Prior to January 2018, perioperative management of patients undergoing endoscopic transsphenoidal pituitary surgery at our hospital was generally the same as other craniotomy patients. Thus, all the patients were arranged in the same ward of neurosurgery, where the inpatient neurosurgical team gave a homogenous management protocol, such as patient education, anesthesia program, and postoperative care. Beginning in January 2018, we had a multidisciplinary team and developed a perioperative management ERAS protocol. Under this approach, all patients admitted for endoscopic transsphenoidal pituitary surgery are routinely arranged in a special pituitary tumor ward. The pituitary neurosurgeon and neuroendocrinologist complete the assessment of pituitary hormones in patients at the time of surgery, the anesthesiologist determines the patient's rapid conscious anesthesia program, and the specialty nurse is responsible for patient education and the implementation of the perioperative program (Table 1).

**Table 1:** Perioperative management protocol of ERAS group.

Multidisciplinary team	Pituitary neurosurgeon
	Neuroendocrinologist
	Anesthesiologist
	Specialty nurse
Preoperative management	Patient education
	Oral carbohydrate loading
	Oral breathing exercise
Anesthetic and surgical management	Rapid conscious anesthesia
	Intraoperative warming
	Keeping the nasal structure intact
	Partial nasal packing
Postoperative management	Keeping nasal ventilation
	Early getting out of bed
	Pain management
	Hormone replacement
	Early postoperative follow-up

During the preoperative management period, oral carbohydrate loading 2 h before surgery and oral breathing exercise are given under the guidance of a specialty nurse. After the operation begins, short-acting anesthetics are used, and body temperature is kept constant. Postoperatively, patients are usually transferred from the recovery room to the pituitary tumor ward without an intensive care unit (ICU) stay unless there are serious complications. On POD1, patients are encouraged to get out of bed early and the catheter is removed as soon as possible. Pain management and hormone replacement evaluation are carried out by the neurosurgeon and neuroendocrinologist on POD1 and the day before discharge. On the day before discharge, patient education concerning how to monitor fluid intake and output, mental state, and epistaxis is given. The first week (1, 3, and 7 days) after discharge, the specialty nurse calls the patients and gives some professional advice according to the patient's different situation. Each patient goes to the neurosurgery clinic for follow-up 1 month after surgery.

### Surgical Technique

Endoscopic transsphenoidal pituitary surgery is performed using a rigid neuroendoscope (length 18 cm; diameter: 2.7 and 4 mm; optics: 0°, 30°, 45°). The right middle turbinate is laterally subluxed in order to establish a surgical passage in the right nostril. Bilateral nasal passages are used when the adenoma is large or belongs to the grade of Knosp 4. The one-nostril, two-hands technique is utilized in most cases combined with two-nostrils, four-hands in a few cases. A mini flap of the nasal septum mucosa is obtained to get material for reconstruction of the sellar floor and to open the access to the sphenoid sinus cavity. After the sellar opening and dural chopping, the tumor removal is performed with the technique of extracapsular resection and using an angled neuroendoscope (optics: 0°, 30°, 45°) to achieve gross total resection. The lumbar drain is not routinely placed at the end of the operation unless there is a high-flow intraoperative cerebrospinal fluid (CSF) leak.

In terms of sellar floor reconstruction and nasal packing, there were significant differences around 2018. In all cases before 2018, the sellar floor was reconstructed with two layers of dural substitute, one positioned inlay and another overlay, and a layer of fibrin glue was used to make the final seal in the end. Finally, a whole piece of dissolvable expansion sponge was placed in the right nasal passage or bilateral nasal passages to reinforce the reconstruction. Beginning in January 2018, a dural substitute positioned inlay and nasal septum bone fragment positioned overlay is used routinely for sellar reconstruction. At the end of the operation, pieces of absorbable NasoPore packs are placed in the sphenoid sinus cavity to buttress the repair. No nasal packing is done in the nasal passages. The septal mucosa is not routinely used for sellar floor reconstruction unless there is a high-flow intraoperative CSF leak in both groups.

### Perioperative and Postoperative Record Analysis

We retrospectively analyzed the records of 78 consecutive patients who underwent endoscopic transsphenoidal pituitary surgery by a single neurosurgeon (Yuehui Ma) in our hospital within 2 years before and after implementation of the ERAS protocol and minimally invasive technique.

Data was collected from inpatient electronic medical records and the outpatient records of the neurosurgeon and endocrinologists. The information collected included general patient information, past medical history, clinical manifestations and signs, and perioperative laboratory values. Detailed information about the patient's operation, including the operation details and complications, could be obtained from the operation report and inpatient records. The results of tumor pathology and imaging examinations were collected, including magnetic resonance imaging (MRI) and computed tomography. The data collected from the inpatient and outpatient records included pituitary hormones and blood electrolyte levels; the doses of glucocorticoids administered before, perioperative, and postoperative; and the treatment of diabetes insipidus (DI). The extent of tumor resection was judged by the MRI 1 month after surgery. Hospital charges (RMB) included fees from admission to the day of discharge. The evaluation of the quality of the early postoperative course is based on the following parameters: nasal ventilation, out of bed, liquid supplement, occurrence of early surgical complications, postoperative headache score, and postoperative hospital LOS. The need for pituitary hormone replacement at the time of discharge and 1 month after operation was recorded. In the early postoperative period and within 30 days after discharge, patient records were checked for emergency visits or hospital readmission.

### Data Analysis

We calculate descriptive statistics, frequency of categorical data, mean ± SD of continuous data and compare the value of the ERAS group with the value of the control group. Continuous variables are compared by Wilcoxon rank sum test. Fisher's exact test was used to compare categorical data. *P* < 0.05 is considered significant. Use SPSS software (version 16.0, SPSS, Inc.) for statistical analysis.

### Ethics Statement

This study was examined and approved by the Ethics Committee of the First Affiliated Hospital of Zhejiang University Medical School. Informed consent on the treatment plan was completed in the talk and signing session before the operation in both the control and ERAS groups. Therefore, there is no need to sign another informed consent for the study considering the retrospective nature of the study and the (emotional) burden that would result from contacting the patients or their relatives to obtain consent.

## Results

### Patient Characteristics

Table 2 shows the characteristics of the ERAS (41 patients) and the control groups (37 patients). There were no differences with regard to age and sex distribution in the two groups. Seven and five patients in the ERAS and control groups had undergone a medical treatment, respectively, three patients in both groups had undergone a prior surgery procedure, and one patient in the ERAS group had been treated with a gamma knife. The preoperative lesion maximal diameter was mostly 1–3 cm, and most of the Knosp classifications belonged to grades 1 and 2 in both groups. Non-functional pituitary tumors were the most common pituitary tumors, 24 and 17, respectively, followed by PRL, GH, and ACTH pituitary tumors. There were no significant differences between the two groups in terms of the operative time, intraoperative CSF leak, and lumbar drain placed after operation. Nasal packing was the biggest difference between the ERAS and control groups (*P* < 0.05). All patients in the control group were fully packed with a whole piece of dissolvable expansion sponge in the right nasal passage or bilateral nasal passages to buttress the repair. Due to high-flow intraoperative CSF leak, only 1 patient in the ERAS group was fully packed; the other 40 patients were partially packed in the sphenoid sinus cavity to reinforce the repair with no nasal packing in the nasal passages.

**Table 2:** Preoperative patient characteristics.

	**ERAS group**	**Control group**	***P*-value**
No. of patients	41	37	n.s.
Age, years, average (range)	49.3 ± 15.3	50.8 ± 8.9	n.s.
Male/female	24/17	16/21	n.s.
**Preoperative condition**			
Medical treatment	7	5	n.s.
Surgery	3	3	n.s.
Gamma knife treatment	1	0	n.s.
**Lesion maximal diameter (cm)**			
Diameter <1	9	5	n.s.
1 < diameter <3	32	31	n.s.
Diameter>3	0	1	n.s.
**Knosp classification**			
0	2	4	n.s.
1	16	12	n.s.
2	15	16	n.s.
3	5	3	n.s.
4	3	2	n.s.
**Pituitary hormone level**			
Non-functioning	24	17	n.s.
PRL	7	10	n.s.
GH	9	8	n.s.
ACTH	1	2	n.s.
TSH	0	0	n.s.
Gonadotropin	0	0	n.s.
Length of surgery (min)	169 ± 69	185 ± 72	n.s.
Intraoperative CSF leak	15	12	n.s.
Lumbar drain placed	1	2	n.s.
Nasal packing			n.s.
Partially packed	40	0	<0.05
Fully packed	1	37	<0.05

*PRL, prolactin; GH, growth hormone; ACTH, adrenocorticotropic hormone; TSH, thyroid-stimulating hormone; CSF, cerebrospinal fluid; n.s., no significance*.

### Early Postoperative Outcomes

**Figure 1:**
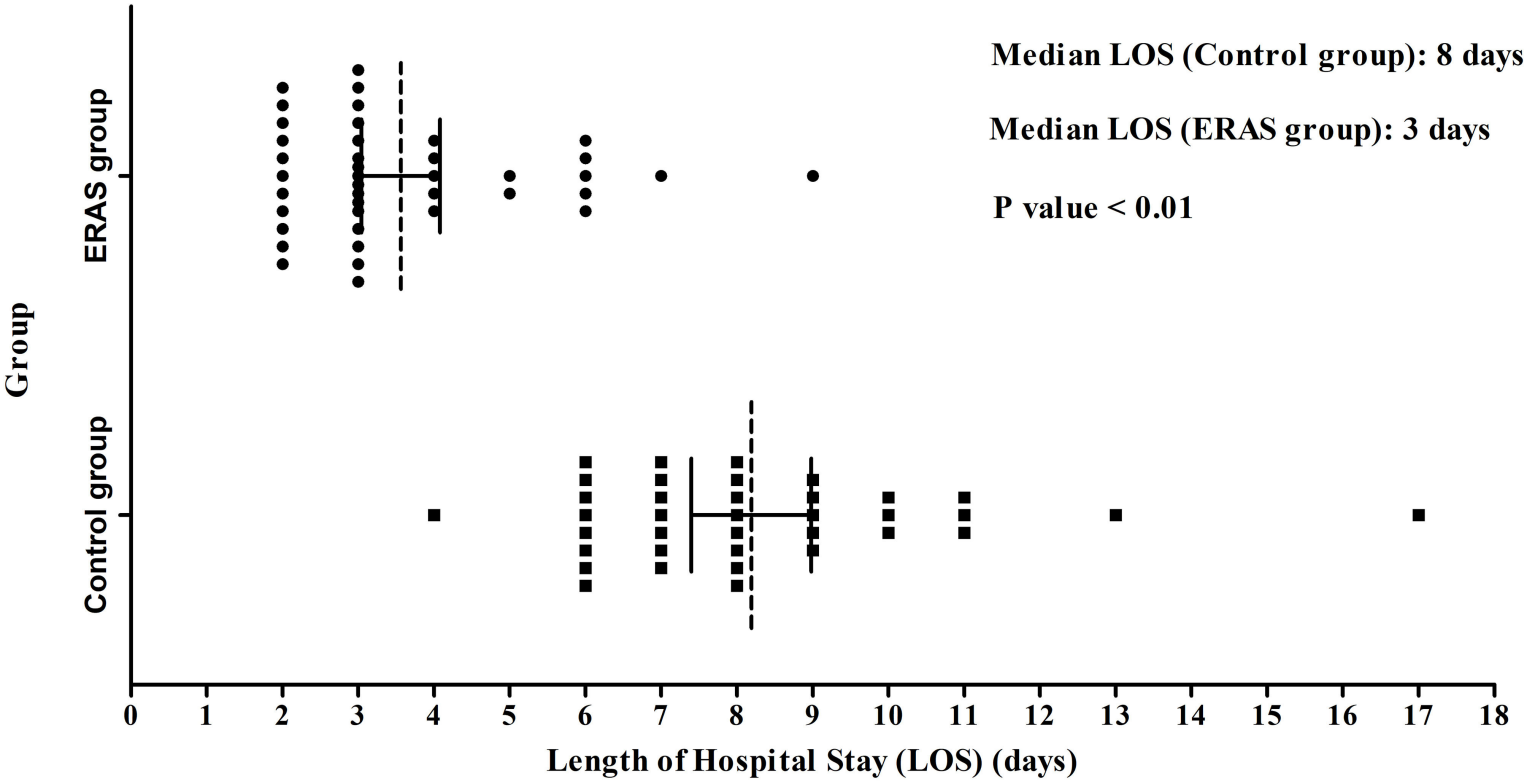
LOS in the ERAS and Control groups. The median LOS of the ERAS group (3 days) was shorter than that of the control group (8 days) (*P* < 0.01). LOS, length of stay after operation.

The postoperative status, especially on POD1, was different between the ERAS and control groups (*P* < 0.05; Table 3). Thirty-four patients in the ERAS group could get out of bed on POD1, and 35 patients had nasal ventilation in the group. There were no patients that could get out of bed and all the patients breathed through the mouth in the control group. The mean headache score was 1.0 ± 0.9 in the ERAS group and 2.1 ± 0.7 in the control group. Ten patients in the ERAS group needed a liquid supplement, and all the patients needed liquid supplement in the control group. The postoperative hospital LOS of the two groups was obviously different with 3.1 ± 1.6 days and 8.2 ± 2.3 days, respectively (*P* < 0.05; Figure 1). The discharge rate on POD1 was as high as 83% in the ERAS group, compared with only 30% in the control group (*P* < 0.05). The ERAS group had a better economic benefit with fewer hospital charges. The mean hospital charge in the ERAS group was 29,720 ± 8,541 yuan vs. 35,879 ± 6,583 yuan in the control group (*P* < 0.05).

**Table 3:** Early postoperative outcomes.

	**ERAS group**	**Control group**	***P*-value**
**Extent of resection (Postoperative MRI within 1 month)**			
Gross total resection	39	34	n.s.
Residual in cavernous sinus	2	3	n.s.
Residual suprasellar	0	0	n.s.
**Postoperative status on POD1**			
Nasal ventilation	35	0	<0.05
Out of bed	33	0	<0.05
Headache score	1.0 ± 0.9	2.1 ± 0.7	n.s.
Liquid supplement	10	37	<0.05
Postoperative hospital length of stay (days)	3.1 ± 1.6	8.2 ± 2.3	<0.05
Patients discharged on POD1 (%)	83% (34)	30% (11)	<0.05
Hospital charges (RMB)	29,720 ± 8,541	35,879 ± 6,583	<0.05
**Complications**			
CSF leakage (Lumbar drain/Operation)	1	2	n.s.
Diabetes insipidus	4	3	n.s.
Fluid and electrolyte abnormalities	8	5	n.s.
Meningitis	0	0	n.s.
**30-Day readmissions**			
Epistaxis requiring operative repair	0	0	n.s.
CSF leakage (Lumbar drain/Operation)	0	0	n.s.
Hyponatremia	1	0	n.s.
**Hormone replacement**			
**Time of discharge**			
hydrocortisone	10	11	n.s.
levothyroxine	3	5	n.s.
DDAVP	2	3	n.s.
**1M**			
hydrocortisone	3	2	n.s.
levothyroxine	2	0	n.s.
DDAVP	0	0	n.s.

*MRI, magnetic resonance imaging; POD, postoperative day; Pre, preoperative; DDAVP, desmopressin acetate hydrate; 1M, 1 month after surgery*.

Endocrine complications after operation were similar in the ERAS and control groups (Table 3). DI was the most common one with 4 and 3 patients in the ERAS and control groups, respectively (*P* > 0.05, Table 3). In the DI cases, two patients in the ERAS group needed DDAVP therapy at the time of discharge and 3 patients in the control group. All the patients in both groups did not need DDAVP therapy at the 1-month follow-up. Fluid and electrolyte abnormalities were the secondary change after DI. In the ERAS group, fluid and electrolyte abnormalities occurred in 8 patients during postoperative hospitalization, and 1 patient required readmission because of hyponatremia within 30 days of discharge. Five patients in the control group had transient fluid and electrolyte abnormalities after operation without readmission. Rates of early postoperative hormone replacement were common in both groups. Ten patients needed hydrocortisone replacement at the time of discharge and 11 patients in the control group. The hydrocortisone replacement was transient; only 3 and 2 patients needed hydrocortisone therapy at the 1-month follow-up. The condition of levothyroxine replacement was similar to hydrocortisone therapy; only 2 patients needed levothyroxine therapy at the 1-month follow-up.

There were no obvious differences concerning the neurosurgical complications between the ERAS and control groups. Only one patient in the ERAS group had intraoperative CSF leakage with lumbar draining after the operation. Two patients needed lumbar draining after the operation because of intraoperative CSF leak in the control group. Meningitis did not occur in both groups.

The extent of resection according to the postoperative MRI within 1 month was similar in the ERAS and control groups (Table 3). Most of the postoperative MRI was done during 3 days after operation. Thirty-nine patients had gross total resection in the ERAS group compared to 34 patients in the control group. There were two patients having residual cavernous sinus in the ERAS group compared to three patients in control group. There was no patient having suprasellar residual in both groups.

## Discussion

The quality of the early postoperative course is the most important factor that influences the early surgical effect after endoscopic transsphenoidal pituitary surgery (11, 12). The current analysis of the results in our study mainly focused on the quality of the early postoperative course and clinical outcomes. The main results after implementation of the minimally invasive endoscopic pituitary surgery and ERAS protocol were a significantly shorter LOS from a median of 8 to 3 days and a more comfortable postoperative status on POD1. In addition, the most important economic impact was a significant reduction in hospital charges and health resource utilization. The reduction of LOS and the implementation of minimally invasive endoscopic pituitary surgery did not reduce the safety of patients because unplanned postoperative visits or readmissions (including epistaxis, CSF leakage, and hyponatremia) within 30 days after surgery did not increase. In our experience, an ERAS protocol for the management of patients undergoing minimally invasive endoscopic pituitary surgery reduces hospitalization duration and improves the quality of the early postoperative course without compromising patient safety.

In the study, all operations were performed by a single experienced professional pituitary neurosurgeon in our department in a relatively short period of time, thus reducing the possibility that surgical or hospital factors may explain the observed decrease in LOS. The most important principle of our minimally invasive endoscopic pituitary surgery is to keep the nasal cavity structure intact during the operation as much as possible and to restore the normal patency of the nasal cavity after the operation. First, the majority of cases are completed using the one-nostril, two-hands technique, which minimizes interference with the normal structure of the nasal cavity. The right middle turbinate is laterally subluxed, and nasal mucosa is fully contracted in order to establish a surgical passage in the right nostril. As we know, the both-nostrils approach provides more freedom of movement and wider exposures, which is very important when the adenoma is large or belongs to the grade of Knosp 4. The one-nostril, two-hands technique is found to be enough in most cases; smaller tumor diameter and lower Knosp grade in our study may be important influencing factors. At the end of the surgery, the right middle turbinate is reset, and the nasal mucosa remains intact. We never remove the middle or upper turbinate because the probability of empty rhinoplasty after surgery is greatly increased, and the chance of postoperative nasal bleeding is increased (13–15). Second, during the reconstruction of the sellar floor, we routinely take a nasal septal bone fragment for bone reconstruction together with the multilayer repair technology. The most important purpose is to provide bone support to the sellar floor, improving the safety of patients getting out of bed on POD1. For cases with significant depression of the saddle septum after tumor resection, bony reconstruction of the sellar floor is particularly important for the safety of patients getting out of bed (16, 17). For cases without CSF leak or tiny diaphragm layer, gelfoam may be sufficient for sellar floor reconstruction, but this way is impossible to ensure safety of discharge on POD1. Third, nasal packing as a rigid support is not as important as before because of the bony reconstruction of the sellar floor. At the end of the operation, pieces of absorbable NasoPore packs are placed in the sphenoid sinus cavity to buttress the bony reconstruction with no nasal packing in the nasal passages. This is very important for improving the patient's postoperative status on POD1, especially the recovery of early nasal ventilation (7).

The neurosurgery ERAS protocol is implemented in selective craniotomy, which confirmed the reduction of postoperative LOS and faster recovery without increasing the incidence of complications (18, 19). The impact of the ERAS protocol on endoscopic transsphenoidal pituitary surgery has not been tested. The preliminary results in our study have confirmed the effectiveness and security of the ERAS protocol, which is needed for further evaluation in large cases studies.

Briefly, the perioperative management ERAS protocol consists of four main sections (Table 1): (1) A multidisciplinary team includes a pituitary neurosurgeon, neuroendocrinologist, anesthesiologist, and specialty nurse. Such a team can improve the effect of endoscopic transsphenoidal pituitary surgery, reduce surgical complications, and promote better perioperative treatment (20, 21). Anesthesiologists joining the multidisciplinary team is the biggest difference from a classic multidisciplinary team, which usually contains a pituitary neurosurgeon and neuroendocrinologist (22, 23). We believe that the anesthesiologist's anesthesia program and perioperative management strategy are important for the implementation of the operation and the recovery of postoperative anesthesia (24). (2) Preoperative management includes patient education, oral carbohydrate loading 2 h before surgery, and oral breathing exercises. The purpose of patient education is to better cooperate with the ERAS protocol and improve patient follow-up compliance after early discharge. The reason why we take oral carbohydrate loading 2 h before surgery is that, compared with regular fasting, oral carbohydrate-rich clear liquid before surgery can reduce insulin resistance and improve perioperative hunger, thirst, and fatigue (25). All patients still needed to fast for 12 h before surgery although patients in the ERAS group received 400 mL of oral carbohydrate loading 2 h before surgery. This protocol is widely accepted in general surgery, and we are also further studying the implementation in other types of neurosurgical procedures. (3) Anesthetic and surgical management includes rapid conscious anesthesia, intraoperative warming, keeping the nasal structure intact, and partial nasal packing. Intraoperative warming is actually part of rapid conscious anesthesia, and their purpose is to improve the quality of postoperative anesthesia resuscitation and avoiding the possibility of an ICU stay after surgery (24). (4) Postoperative management includes keeping nasal ventilation, early getting out of bed, pain management, hormone replacement, and early postoperative follow-up. Nasal ventilation is an important factor affecting the quality of the early postoperative course after endoscopic transsphenoidal pituitary surgery. Keeping the nasal structure intact and partial nasal packing is the key to ensuring postoperative nasal ventilation.

However, our research has some limitations that may limit generalization. The number of cases in this study is limited, and our patients have a wide range of ages, preoperative conditions, and pituitary hormone levels. These differences coupled with the range in lesion diameter and Knosp classification do not make clear which patients are really suitable for early discharge in clinical practice. Additionally, the application of the concept of ERAS in neurosurgery is later than general surgery. The successful implementation of pituitary tumor day surgery requires close cooperation between neurosurgeons, endocrinologists, anesthetists, and a specialist nurse, which may require specialized support staff and patients who can strictly follow instructions. For example, educating patients to discharge early is a challenging task because most neurosurgery patients have difficulty receiving discharge on POD1. Finally, the end time of our cases is 1 month after surgery. The long-term prognosis of patients and whether patients were satisfied with early discharge need to be confirmed by further follow-up. Despite these limitations, we believe our results suggest that improvement of the quality of the early postoperative course after endoscopic transsphenoidal pituitary surgery is an achievable goal by improving the surgical technique and perioperative management strategy.

## Conclusion

This study demonstrates that the quality of the early postoperative course can be improved when a neurosurgical ERAS protocol and minimally invasive endoscopic transsphenoidal pituitary surgery are implemented. Preserving the normal nasal structure and restoring the nasal cavity can significantly improve the patient's postoperative comfort, and the ERAS protocol improves the safety of early discharge. Endoscopic transsphenoidal pituitary day surgery could be recommended in some classes of patients though further evaluation in large cases studies is warranted.

## Data Availability Statement

The datasets generated for this study are available on request to the corresponding author.

## Ethics Statement

The studies involving human participants were reviewed and approved by Ethics Committee of the First Affiliated Hospital of Zhejiang University Medical College. Written informed consent for participation was not required for this study in accordance with the national legislation and the institutional requirements.

## Author Contributions

XP, YM, and RZ contributed to study design. XP, MF, JJ, and JS contributed to data collection. XP, YM, and JJ contributed to analysis and interpretation of the data. XP contributed to manuscript drafting. RZ and YM contributed to manuscript revising and responsibility for conduct of research and final approval. All authors contributed to the article and approved the submitted version.

## Conflict of Interest

The authors declare that the research was conducted in the absence of any commercial or financial relationships that could be construed as a potential conflict of interest.
